# Comparison of education group strategies and home visits in type 2 diabetes mellitus: clinical trial ^1^

**DOI:** 10.1590/1518-8345.2315.2979

**Published:** 2017-12-21

**Authors:** Jéssica Caroline dos Santos, Daniel Nogueira Cortez, Maísa Mara Lopes Macedo, Edna Afonso Reis, Ilka Afonso Reis, Heloísa Carvalho Torres

**Affiliations:** 2 Master’s student, Escola de Enfermagem, Universidade Federal de Minas Gerais, Belo Horizonte, MG, Brazil. Scholarship holder at Coordenadoria de Aperfeiçoamento de Pessoal de Nível Superior (CAPES), Brazil.; 3 PhD, Adjunct Professor, Universidade Federal de São João del Rei, Divinópolis, MG, Brazil.; 4 MSc, RN, Hospital da Clínicas, Universidade Federal de Minas Gerais, Belo Horizonte, MG, Brazil.; 5 PhD, Associate Professor, Departamento de Estatística, Universidade Federal de Minas Gerais, Belo Horizonte, MG, Brazil.; 6 PhD, Adjunct Professor, Departamento de Estatística, Universidade Federal de Minas Gerais, Belo Horizonte, MG, Brazil.; 7 PhD, Associate Professor, Escola de Enfermagem, Universidade Federal de Minas Gerais, Belo Horizonte, MG, Brazil.

**Keywords:** Health Education, Self Care, Diabetes Mellitus, Home Visit, Primary Health Care, Clinical Trial

## Abstract

**Objective:**

to compare the adherence and empowerment of patients with type 2 diabetes mellitus for self-care practices and glycemic control in group education strategies and home visits.

**Method:**

Clinical trial with ten randomized clusters, performed with 238 patients with type 2 diabetes mellitus distributed in group education, home visit, and control group. Socio-demographic data, glycated hemoglobin and those obtained from the self-care and empowerment questionnaires were collected. Statistical analysis was performed separately by educational strategy.

**Results:**

the mean age of the patients was 57.8 years old (SD = 9.4 years old), with a predominantly female participation (66.4%). Both strategies presented similar results regarding adherence to self-care practices and patient empowerment. There was also a reduction in glycated hemoglobin levels; however, only in the education group, the difference presented statistical significance (p <0.001).

**Conclusion:**

the strategies were effective; however, group education presented better glycemic control results in relation to the home visit. International registry: NCT02132338 and national: RBR-92j38t in the clinical trials registry.

## Introduction

Type 2 diabetes mellitus (DM2) is 90% of the diagnoses of this chronic condition. It is a global health problem due to its high incidence and it is related to inadequate self-care behaviors, such as sedentary lifestyle and inappropriate diet. It is estimated that there are 415 million people in the world aged between 20 and 79 years old who have this condition and the expectation is that this number increases progressively, reaching 642 million in 2040. In Brazil, 14.3 million individuals have this diagnosis^1^ ^-^ ^2^.

As a way of collaborating in activities that promote control of this chronic condition, educational strategies such as group education and home visits have presented positive results, aiming at self-care practices in type 2 diabetes mellitus, in the context of adequate nutrition, physical exercise (3), and capacity for problem solving, among other things. When based on the approach of empowerment, through dialogue, patient appreciation, knowledge, and attitudes these strategies are considered effective in promoting and preventing complications^4^ ^-^ ^5^.

For this study, self-care was defined as the actions that patients take to lead a healthy lifestyle for their own well-being and health, such as the adoption of concrete behaviors of self-medication, healthy eating, and physical exercise. In this perspective, the empowerment approach supports self-care education in DM2 and stimulates the autonomy of the patient. Also, the literature indicates that the qualified and intentional involvement of the patient to make decisions is effective in coping with this chronic condition^3^ ^-^ ^5^. It is believed that group education and home visits based on an accessible and emancipatory education that favors problematization, the construction of knowledge and skills, as well as the approach to empowerment, can influence behavior change and encourage the patient self-care practices^2^ ^,^ ^5^ ^-^ ^6^.

However, there is little research that evaluates the effectiveness of educational strategies in primary health care^2^ ^,^ ^6^. According to previous studies, the existing findings are incipient and heterogeneous regarding educational interventions and study samples, and there is no single standardized program to reach patients with diabetes^7^ ^-^ ^9^. Another study comparing educational strategies for this public, proposes the continuity of research of this nature, aiming to understand the threshold between individual and group strategies, considering this process as dynamic and requiring continuous evaluation^10^.

Based on the above, the DM2 empowerment education program, developed by the School of Nursing of the Federal University of Minas Gerais (EEUFMG) in primary health care in the city of Divinópolis (MG), used home visit and education group strategies to promote adherence to self-care practices and patient empowerment, aiming at improving glycemic control.

The aforementioned DM2 empowerment program was a 12-month randomized clinical trial that included group education strategies, home visits, and telephone intervention support when needed. These strategies were selected because it was believed that together they could achieve a greater diversity of patients with this chronic condition, promoting the improvement of self-care and glycemic control. The study was conducted by a team of nurse researchers, with the support of a nutritionist and physiotherapist. The patients who participated in the intervention were compared with the patients who received only usual care performed by the health services. However, to date, these strategies have not been analyzed independently by the educational program^2^.

Thus, this study aimed to compare the adherence and empowerment of patients with type 2 diabetes mellitus for self-care practices and glycemic control in group education strategies and home visits.

## Method

A clinical trial was conducted with randomized clusters involving 238 patients with type 2 diabetes mellitus treated in ten family health strategies (ESF) of primary health care in the city of Divinópolis (MG), which concluded participation in the diabetes empowerment program, from December 2014 to January 2016.

For the systematization of the educational interventions and the setting of this study, the ten family health strategies (ESF) of the municipality with the highest number of DM2 patients were selected, so each ESF was considered a cluster.

The sample size calculation considered the cluster effect^11^. The value of the intra-class correlation coefficient was estimated at r = 0.008, taking previous studies with similar populations as a reference^12^ ^-^ ^13^. The sample also used: α = 0.05 (level of significance); ω = 0.90 (test power); *d* = 1 (standardized effect on the dependent variable), *n* = 80.9 (average size of clusters), N = 1320 (total population) and k = 10 units of the ESF (clusters). For each large study group (control group - CG and intervention group - IG), a minimum number of 65 patients was determined. Considering 35% as a value for the friction rate, the minimum number at the beginning of the study should be 100 patients in each group.

Through a lottery carried out in the *R* (2015)^14)^ environment, three ESFs were allocated to group education (93 patients), two for the home visit (34 patients) and five ESFs were allocated to the control group (111 patients). After allocation of the ESF to the strategies, the comparison groups were found to be homogeneous in terms of education level and glycated hemoglobin. The division of the five ESFs that would receive the intervention between the home visit and group education considered that the home visit is an educational strategy operationally more expensive than the group education.

Randomization by cluster rather than by individuals was chosen because it allowed a better operationalization of the study and to avoid that the contact between individuals attended by the same team, but belonging to different educational strategies, could bias the results obtained^15^.

The inclusion criteria for participation in the research consisted of having type 2 diabetes mellitus, age between 30 and 80 years old and willing to participate in group education and receive a home visit. Chronic DM2 complications (defined as nephropathy, retinopathy, limb amputation and diabetic foot) and the patient’s refusal to participate in the study were established as exclusion criteria. Patients who participated in less than 6 group education meetings and less than 4 home visit meetings were discontinued from the study.

This study complied with ethical standards in research, and it was approved by the Research Ethics Committee involving Human Subjects of the Federal University of Minas Gerais (COEP/UFMG, protocol 426.968/2013). Participants were clarified about the study and its confidentiality. After the acceptance, all of them signed the Free and Informed Consent Form (TCLE) in two copies. The registration number in the international clinical trials registry is NCT02132338 and, in the national registry, RBR-92j38t, and followed all CONSORT (Consolidated Standards of Reporting Trials) guidelines^16^.

Educational strategies focused on adherence and empowerment for self-care in type 2 diabetes mellitus worked through the behavior change protocol and addressed the following items: 1) exploration of the problem; 2) feelings and emotions; 3) feeding, with emphasis on feeding frequency and fiber intake; 4) nutrients (carbohydrates, proteins, fats, vitamins, and minerals); 5) reading of food labels; 6) benefits of physical activity and 7) complications of type 2 diabetes mellitus^12^. The strategies were conducted by health researchers (five nurses, a nutritionist, and a physiotherapist), the ESF professionals collaborated with the availability of the DM2 patient registry, providing and indicating locations for the development of the group, and some as interlocutors between the researcher and the participant in the study.

**Figure 1:**
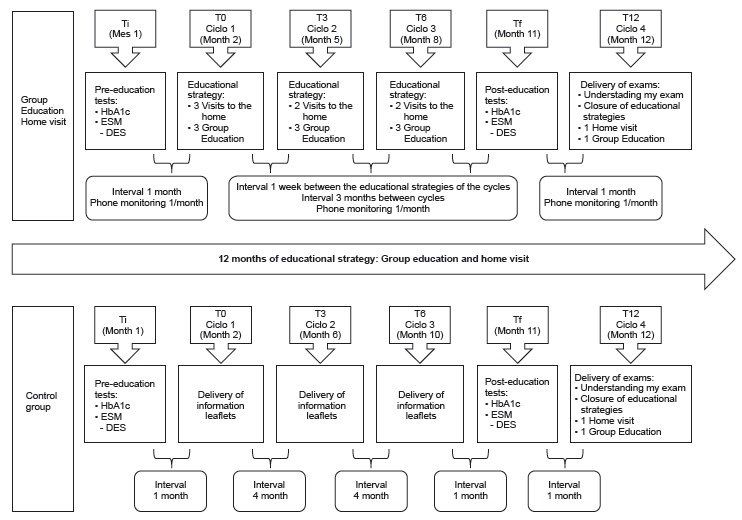
Cycle development stages.

Source: Elaborated and adapted from Cortez (2016).

Group education and home visits occurred in the 12-month period, through six times and four cycles, enumerated as follows: initial time (Ti), with the application of pre-education tests; time 0 (T0) with cycle 1; time 3 (T3) with cycle 2; time 6 (T6) with cycle 3, periods in which the strategies were developed; (Tf), with post-education tests and time 12 (T12) with cycle 4, in which a single meeting was held for explanations and delivery of the glycated hemoglobin result, as shown in Figure 1.

Between the cycles, there was an interval of three months. The number and duration of the meetings of each cycle were established according to the specificity of each strategy. During the intervals between the cycles of both group education and home visits, monthly telephone monitoring was done to address the possible doubts of patients with diabetes that arose during this period and to strengthen self-care practices.

Group education had 10 meetings in all, with cycles 1, 2 and 3 having three meetings each and cycle 4 only one meeting. Each meeting had the average participation of 10 patients and lasted approximately 120 minutes, being conducted by at least two professionals: a facilitator and a support professional. The participants were arranged in a circle so they could form a conversation circle. As a trigger for the discussions and to stimulate the participation of all, were used dynamics and interactive activities. Each meeting of the cycle was offered three times, in distinct days and periods to reduce the chance of loss of the patient. Balanced snacks and fruits were served during the meetings to stimulate healthy eating.

The home visit had 8 meetings, which happened as follows: three meetings in cycle 1; two meetings in cycles 2 and 3 and a meeting in cycle 4. The strategy was conducted by two professionals: a facilitator and a support professional. Each meeting had an average duration of 90 minutes and the scheduling of the visit was done according to the patient’s availability. If there was an impediment after scheduling, another day was offered for the meeting, including at night and on weekends.

Participants in the control group participated in the educational practices developed in the routine of the respective health units and maintained the conventional follow-up, performed in the Family Health Teams, through clinical care. These patients received two telephone calls to maintain the link and reduce losses, to confirm the participation of patients as control and received two semiannual meetings to deliver leaflets, without the direct intervention of the researchers.

Questionnaires were used to collect sociodemographic data at the initial time (Ti). Also, instruments were used to measure adherence and empowerment for self-care for type 2 diabetes mellitus. A glycated hemoglobin test was also performed to be used as a clinical indicator. Glycated hemoglobin and the instruments related to adherence and empowerment for self-care were also applied in two moments: at the initial time (Ti), before the beginning of the educational strategies, and at the final time (Tf). The collection was done through semi-structured interviews, conducted by the study researchers themselves in a quiet and reserved environment, and these professionals were also responsible for applying educational strategies.

For the sociodemographic characterization of the patients, a questionnaire was elaborated to collect data of the following variables: gender, categorized as “female” or “male”; age, self-reported, in years; marital status, self-declared and later categorized as “with partner” or “without partner”; education level, self-declared and later categorized into “incomplete elementary school” and “complete elementary school through post-graduation”; occupation, self-declared and later categorized as “active” or “inactive”; and disease time, categorized as “0 to 4 years,” “5 to 10 years,” or “over 10 years.”

Self-care adherence was measured through the Self-Care Questionnaire in Diabetes Mellitus (ESM), which consists of eight closed questions about self-care behaviors related to diet and physical exercise adopted in the seven days prior to the instrument’s collection. The ESM questionnaire is parameterized in two ways, depending on the item to be answered: the first form is in relation to the number of days of the week, from zero to seven; the second form used is a scale governed by the occurrence of behavior, categorized as “never,” “rarely,” “sometimes,” “usually,” and “always.” For analysis, the sum of the alternatives of each item totals one point, and the instrument has a total score of eight points. In items that evaluate the consumption of high fat and sweet foods, the values ​​are reversed. The patient is considered to have adhered to a change in behavior if he or she achieves a minimum score of five points or if there is an increase in the score between before and after educational strategies ^6^.

The empowerment was measured by the Brazilian version of the Diabetes Empowerment Scale-Short Form (DES-SF) (17). This instrument contains eight affirmations with which the respondent should identify some level of agreement using a five-point Likert scale, which starts from “totally disagree” (1 point) and goes “totally agree” (5 points). The overall score is given by the average grade of each of the eight items. For the measurement of empowerment, the following score was considered: low, from 1 to 2.3; mean, from 2.4 to 3.7; and high, from 3.8 to 5.0^18^.

As a clinical variable, glycated hemoglobin (HbA1c), a marker used to evaluate glycemic control in people with type 2 diabetes mellitus, was used. For this study, it was considered the reference value for good control of DM2 if HbA1c ≤ 7%, parameter internationally recommended^1^.

The descriptive analysis was performed by frequency calculations for categorical variables and measures of central tendency (mean and median) and dispersion (SD: standard deviation) for the quantitative variables. Statistical analyses were performed in the SPSS-Statistical Package for the Social Sciences (version 20.0). The Shapiro-Wilk test was used to verify the normality for the distribution of probabilities of the dependent variables.

To verify if the groups of participants were similar in relation to sociodemographic and clinical variables prior to the strategies, the ANOVA test was used for the comparison of means and the chi-square test for the comparison of proportions.

For the intra-group and inter-group comparisons, paired Student’s t-tests and for independent samples or their non-parametric counterparts (Wilcoxon and Mann-Whitney, respectively) were used. In all tests, the results with p <0.05 were considered statistically significant.

The three groups were compared in relation to the variables HbA1c, empowerment, and level of self-care. The relative effect (Δ) on a variable was defined as the difference between its values in the initial period and the final period, divided by the initial value. The values found were multiplied by 100 to transform it into percentage variations^2^.

## Results

**Figure 2:**
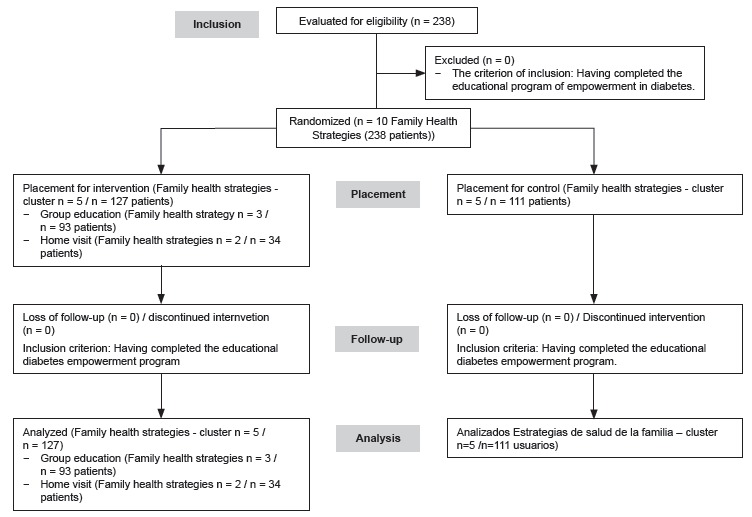
Flow diagram of the progress of clusters and individuals by phases of the randomized trial.

There were 111 comprised the control group (46.6%), 93 (39.1%) from the group education strategy and 34 (14.3%) from the strategy home visit of the 238 diabetes patients who completed the empowerment program. Following the randomized trial guidelines^14^, Figure 2 shows a flow diagram of the progress of clusters and individuals by phases of the randomized trial.

Regarding the sociodemographic characteristics evaluated, the mean age was 57.8 years old (SD = 9.4 years old); greater female participation, with 158 patients (66.4%); 181 (78.1%) had a partner; 163 (68.5%) had at least complete primary education; 128 (53.8%) had no occupation; and 167 (70.16%) reported the time of diagnosis of DM2 greater than 5 years.

The sociodemographic characterization was performed for the control group and the strategies of group education and home visit, separately. The results demonstrate homogeneity (p> 0.05) of the sociodemographic variables in the baseline of all three study groups (CG, EG, HV), however, a significant difference was observed in relation to the disease time variable, thus, that the groups were not statistically different in most of the variables used and making possible the post-intervention comparisons. (Table 1).

**Table 1:** Description of sociodemographic variables of patients with type 2 diabetes mellitus, participants of the control (CG), group education (EG) and home visits (HV), primary care in the city of Divinópolis (MG), Brazil, 2016

**Variable**		**Total** **(n=238)**	GC* **(n=111)**	**EG** ^**†**^ **(n=93)**	**VHV** ^**‡**^ **(n=34)**	***P*** ^**§**^
**Age, in years (mean ± SD||)**		**57,8 ± 9,4**	**57,5 ± 9,7**	**59,2 ± 8,5**	**54,9 ± 10,5**	**0,600** ^**¶**^
**Gender (n (%))**						
	**Male**	**80 (33,6)**	**38 (34,2)**	**34 (36,6)**	**8 (23,5)**	0,38**
	**Female**	**158 (66,4)**	**73 (65,8)**	**59 (63,4)**	**26 (76,5)**	
**Education level (n (%))**						
	**Up to incomplete elementary school**	**163 (68,5)**	**73 (65,8)**	**67 (72,1)**	**23 (67,6)**	0,630**
	**Complete Elementary school post-graduation**	**75 (31,5)**	**38 (43,2)**	**26 (27,9)**	**11 (32,4)**	
**Marital status (n (%))**						
	**With a partner**	**181 (78,0)**	**87 (78,4)**	**66 (70,9)**	**28 (82,4)**	0,300**
	**Without a partner**	**57 (24,0)**	**24 (21,6)**	**27 (29,1)**	**6 (17,6)**	
**Occupation (n(%))**						
	**Active**	**110 (46,2)**	**55 (49,5)**	**38 (40,8)**	**17 (50,0)**	0,410**
	**Inactive**	**128 (53,8)**	**56 (50,5)**	**55 (59,2)**	**17 (50,0)**	
**Time of Disease (n(%))**						
	**0 to 4 years**	**71 (29,8)**	**21 (18,9)**	**34 (36,5)**	**16 (47,0)**	<0,001**
	**5 to 9 years**	**167 (70,2)**	**90 (81,1)**	**59 (63,5)**	**18 (53,0)**	

*GC: Control Group. †EG: |Education group. ‡VD: Home visit. §p: p-value: level of significance. ||SD: standard deviation. ¶Test ANOVA. **Chi-square test.

The Shapiro-Wilk test showed that the distribution of the variables HbA1c, empowerment, and self-care level cannot be considered Normal (p <0.05). Thus, in each variable, the Wilcoxon test was used to test if the medians of the differences between the initial and final times are equal to zero, separately and within the two educational and control strategies.

Thus, Table 2 shows the results of the clinical variable (HbA1c) and the responses of the DES-SF and ESM questionnaires, at the baseline and after the intervention strategies. It was verified that the patients with DM2 submitted to group education presented a significant improvement in the studied variables. However, no significant reduction of glycated hemoglobin was observed in the diabetic patients who were part of the home visit.

**Table 2:** Mean (minimum and maximum) values of glycated hemoglobin and responses to ESM* and DES-SF questionnaires, and comparison of intragroup medians between baseline (before) and after intervention (after) with type 2 diabetes mellitus of the primary care of the city of Divinópolis (MG), Brazil, 2016

**Variables**	**Control Group**				**Education Group**				**Home Visit**		
	**Before**	**After**	***p*** ^**‡**^		**Before**	**After**	***p*** ^**‡**^		**Before**	**After**	***p*** ^**‡**^
**HbA1c** ^**§**^	**7,40** **(5-14,4)**	**7,40** **(4,9-13,9)**	**0,3000**		**7,80** **(5-7,13)**	**7,10** **(5-12,4)**	**0,0000**		**7,50** **(5-13,5)**	**7,00** **(5,4-13-7)**	**0,9900**
**ESM** ^*^	**3,21** **(1-6,75)**	**3,00** **(1,25-6,1)**	**0,9700**		**3,25** **(1-7,5)**	**4,05** **(1,75-6,25)**	**0,0001**		**3,18** **(1,75-6,25)**	**5,00** **(2,73-6,25)**	**0,0001**
**DES-SF** ^**†**^	**3,64** **(2,71-4,86)**	**4,00** **(2,5-4,88)**	**0,0000**		**3,68** **(2,68-4,71)**	**4,13** **(2,75-5)**	**0,0000**		**3,73** **(2,79-4,46)**	**4,25** **(3,5-4,875)**	**0,0000**

* ESM: Self-care questionnaire for type 2 diabetes mellitus. †DES: Empowerment questionnaire for type 2 diabetes mellitus. ‡p-value: Wilcoxon test for medians of before-after differences. §HbA1c: Glycated hemoglobin.

Regarding the intergroup comparison of glycated hemoglobin results and adherence and empowerment for self-care in type 2 diabetes mellitus, it is seen that both educational strategies contributed to the improvement of adherence and empowerment for self-care. However, group education when compared individually with the control group and the home visit was the strategy that presented the best result in glycated hemoglobin (Table 3).

**Table 3:** Intergroup comparison of the relative effect* on glycated hemoglobin and ESM † and DES-SF ‡ questionnaire responses, between the baseline (before) and after intervention (after), of primary type 2 diabetes mellitus patients of the municipality of Divinópolis (MG), Brazil, 2016

**Time of the Disease**	**Variables**	Relative Effect* (median, %)				**HV** ^**§**^ **x EG** ^**||**^ **x GC** ^**¶**^		**HV** ^**§**^ **x EG** ^**||**^		**HV** ^**§**^ **x GC** ^**¶**^		**EG** ^**||**^ **x GC** ^**¶**^
		**HV** ^**§**^ **n = 16**	**EG** ^**||**^ **n = 21** ****	**GC** ^**¶**^ **n = 34** ****		***p*** ^**^		***p*** ^**††**^		***p*** ^**††**^		***p*** ^**††**^
**0 to 4 years** **(n=71)**	**HbA1c** ^**‡‡**^	**-2,34**	**-6,82** ^**§§**^	**0**		**0,0182**		**0,2494**		**0,4233**		**0,0077**
	**ESM** ^**†**^	**-21,90** ^**§§**^	**-12,84** ^**§§**^	**0**		**0,2991**		**-**		**-**		**-**
	**DES** ^**‡**^	**-18,48** ^**§§**^	**-12,77** ^**§§**^	**-7,82** ^**§§**^		**0,1164**		**-**		**-**		**-**
												
		**VD** ^**§**^ **n = 18**	**EG** ^**||**^ **n = 58**	**GC** ^**¶**^ **n = 90**								
**5 years or more** **(n=167)**	**HbA1c** ^**‡‡**^	**3,3**	**-5.48** ^**§§**^	**0,68**		**< 0,0001**		**0,0062**		**1**		**< 0,0001**
	**ESM** ^**†**^	**57,91** ^**§§**^	**19,84** ^**§§**^	**0,66**		**< 0,0001**		**0,0126**		**<0,0001**		**0,0461**
	**DES** ^**‡**^	**15,12** ^**§§**^	**10,00** ^**§§**^	**9,89** ^**§§**^		**0,1276**		**-**		**-**		**-**

*The *relative effect* (Δ) on a variable was defined as the difference between its values in the final period and initial period, divided by the initial value, and multiplied by 100 (percentage change). †ESM: Self-care questionnaire for type 2 diabetes mellitus. ‡DES: Empowerment questionnaire for type 2 diabetes mellitus. §HV: Home visit. EG: Group education. ¶GC: Control groups. **Kruskall-Wallis test. ††Dunn test with p-values adjusted by the Bonferroni correction. ‡‡HbA1c: Glycated hemoglobin. §§p <0.05 (Wilcoxon’s test).

Also, through the results presented in table 3, it was observed that, in relation to glycated hemoglobin, the patients with less time of illness and who received the education in group obtained the effect statistically different from zero and different from the effect in the control group, with advantage for group education. The effects of different educational strategies (home visit and group education) were not considered statistically different. However, even for glycated hemoglobin, patients with longer disease times also had the effect on group education statistically different from zero. However, a difference was observed not only in the effect of the control group but also in the home visit, with advantage for group education, so that the effects in the home view and in the control group were not considered statistically different.

Adherence to self-care was also analyzed in relation to the time of illness, and the effects of group education and home visit were considered statistically different from zero for patients with longer disease duration and patients with lower disease duration. However, only among the patients with the longer time of illness, a difference was detected between the three groups of the study, with advantage for the home visit.

Regarding empowerment, both for patients with shorter illness times and those with longer illnesses, the effects on group education, home view, and control group were considered statistically different from zero, but no difference between the three groups was captured by the Kruskal-Wallis test.

## Discussion

The data of this study show that the strategies of group education and home visits were presented as an important environment for the improvement of indicators related to adherence and empowerment for self-care practices in type 2 diabetes mellitus after one year of follow-up. These findings corroborate the results of other studies, which also pointed to the effectiveness of these strategies in providing the patient with competencies for health care as the capacity to make conscious decisions, to have autonomy and to reflect on their experience of living with diabetes^(8, 19-21)^.

Group education has been shown to be effective in improving variables, adherence, and empowerment for self-care practices. It was observed that the characteristics of this strategy, such as socialization among peers, exchanges of experience and shared construction of knowledge, reinforced the development of the patients’ co-responsibility in relation to their own health, stimulating the development of self-care and consequently improving glycemic control^(20, 22)^.

It should be mentioned that in group education, the value of peer interaction on living with type 2 diabetes mellitus stands out, and it leverages this educational strategy to a different level when compared to individual strategies, such as home visits. Because the possibility of experiencing situations common to DM2 with other people alleviates the burden of having a chronic condition, reduces the social distance caused by the required self-care practices and offers relational conditions to think about new perspectives of life. All these aspects combined favor better outcomes in adherence and empowerment for self-care and glycated hemoglobin^23^.

The home visit also improved the results of the measures of empowerment and adherence to self-care. This improvement confirms other changes in similar studies. A study carried out with patients with type 2 diabetes mellitus, attended at basic health units of Belo Horizonte, found that a systematic home visit that considers the needs of the patient stimulates adherence to self-care (24). Moreover, a study on educational interventions for patients with diabetes in supplementary care showed that individual follow-up, made possible by the visit, can provide autonomy for the control of diabetes, which favors the reduction of the impact caused by the chronic condition^10^.

During this study, empowerment was used in group strategies and home visits focusing on the patient, aiming at him to assume his responsibilities and help him to define the most appropriate therapy, improving the management of self-care and of glycemic control^25^ ^-^ ^26^. Participating patients demonstrated that they were actively involved in the decision-making process, building and developing goals to achieve satisfactory results in controlling diabetes.

These results also corroborate those presented in an educational program in diabetes, which, due mainly to the interaction and participation of the patients, obtained effective results in improving self-care practices and metabolic control of type 2 diabetes mellitus, confirming the results of this study^27^. In a complementary way, there is a study about the empowerment in adherence to the therapeutic regimen in people with diabetes, carried out in Portugal. In this study, it was found that the majority of participants with a high level of empowerment obtained greater therapeutic adherence to the treatment of diabetes. In other words, the greater the incentive to patient empowerment, the greater will be their adherence to self-care practices^25^.

According to the authors, educational strategies based on empowerment that aim at patient involvement and their co-responsibility for self-care may reinforce the control of this chronic condition (27). Once empowered, patients’ behavioral changes, propitiated by this approach, can extend to subsequent years, ensuring continuity of care for this condition^28^.

Another study conducted with 295 people with type 2 diabetes mellitus in Taiwan, demonstrated that using the empowerment approach to manage this condition can improve knowledge and self-efficacy of the patient that is a belief in their ability to good therapeutic behavior. So, by working with this variable, it is possible to modify life habits, culminating in the improvement of glycemic control^29^.

Regarding adherence to self-care, the ESM questionnaire identified an improvement in both educational strategies, through the adoption of positive behaviors for the control of type 2 diabetes mellitus, such as healthy eating and physical exercise. These results are in agreement with studies that point to group education and home visit as important strategies in the self-care awareness of this condition^29^. However, there are also studies that point out that for these educational strategies to be effective, a commitment of the patient, as well as a proactive and prepared team, is important^8^.

Besides the variables mentioned above, glycated hemoglobin was also an important indicator of self-care behaviors mediated by the empowerment approach. In this study, there was a significant decrease in HbA1c in the group education strategy. However, the home visit did not improve this indicator, which may be related to the fact that contact time was lower than that of group education. In a study about the contact time in educational practices in type 2 diabetes mellitus, it is suggested that educational strategies that total 12 hours of duration are more effective in achieving better results^30^.

The control group that received the traditional follow-up offered by the ESF, did not show improvement in the self-care adherence and glycated hemoglobin variables. However, the empowerment variable showed a statistically significant improvement and this result can be understood as a change in the paradigms of public health services. Studies show that professionals are being encouraged to review their practices and knowledge about this issue since there is an increase in chronic non-communicable diseases in the Brazilian population^31^. This new context may have contributed to the reflection of professionals on the need to rethink the educational strategies developed^32^.

When facing publications of the same nature, this study demonstrated the importance of well-structured educational strategies for both group education and home viewing. Moreover, the way in which the methodology of educational strategies was delineated allows the replication of these strategies in the real conformation of primary health care to Brazilian health^4^ ^,^ ^9^ ^-^ ^10^.

One limitation of this study is that the cognitive and/or intellectual capacity of the patients was not considered, even if they were participants with a wide age group. Also, the need to make the comparisons considering disease time, due to the inhomogeneity of the groups in relation to this variable, reduced the sample sizes in some cases and, consequently, the power of the statistical tests used.

Another limitation that may have occurred is in the place where the study was performed, a city in the interior of Minas Gerais, which has very own sociodemographic characteristics. In the future, it is suggested to replicate this study in a multicentric way or in metropolitan regions.

## Conclusion

The strategies were effective, and group education presented better results in relation to the home visit for adherence and the empowerment of the patient with type 2 diabetes mellitus for self-care and glycemic control practices.
